# Mitral commissurotomy through the left ventricle apical orifice with Heart Ware left ventricular assist device implantation

**DOI:** 10.1186/1749-8090-8-147

**Published:** 2013-06-08

**Authors:** Prashant Nanasaheb Mohite, Bartlomiej Zych, Aron Frederik Popov, Nikhil Patil, Suvitesh Luthra, Mike Hedger, Andre Ruediger Simon, Mohammed Amrani

**Affiliations:** 1 Department of Cardiothoracic Transplantation & Mechanical Support, Royal Brompton and Harefield NHS Trust, Harefield, Hill End Road, London UB9 6JH, UK; 2 Department of Cardiothoracic and Vascular Surgery, Georg-August-University of Goettingen, Robert Koch Strasse 40, Goettingen 37075, Germany; 3 Postal Address: Harefield Hospital, Middlesex UB9 6JH, UK

**Keywords:** Mitral Commissurotomy, Hypertrophic Obstructive Cardiomyopathy, Ventricular Assist Device

## Abstract

Diseased, replaced or repaired mitral valve can lead to restricted blood flow to left ventricle and inadequate flow in left ventricular assist device (LVAD). A middle age woman with ‘burnt out’ hypertrophic cardiomyopathy had mitral valve repair for mitral regurgitation. She needed LVAD to support severe decompensating heart failure. Repaired mitral valve posed a risk of restricted flow through the device. Mitral commissurotomy was performed on beating heart through the left ventricular apical hole created for insertion of inflow cannula of LVAD.

## Background

Left ventricular assist devices (LVADs) offer valuable therapeutic option for patients with end-stage heart failure, either as a short-term support, as bridge to transplant, bridge to recovery or as destination therapy [1]. Mitral valve disease pose a challenge during LVAD implantation as diseased or previously repaired mitral valve can restrict flow to left ventricle (LV) and in turn to LVAD. Routinely, mitral commissurotomy needs complete cardiopulmonary bypass (CPB) with bicaval cannulation, cardioplegic arrest of heart and trans-septal or left atrial access to the valve. Authors demonstrate commissurotomy on beating heart through LV apical orifice for ventricular assist device.

## Case presentation

A 44 year old lady presented with shortness of breath on minimal exertion and swelling over the legs. She was diagnosed of hypertrophic obstructive cardiomyopathy in her early twenties which was successfully treated with trans-catheter septal ablation and she could manage two pregnancies thereafter. She was apparently free of symptoms till last couple of years ago, when she started having dyspnoea on exertion and pedal oedema which worsened rapidly. Echocardiography showed severe mitral regurgitation with dilated and poorly functioning left ventricle for which she underwent mitral valve repair. Her symptoms were not relieved with the surgery and she was classified as decompensating heart failure in NYHA stage three. Echocardiography showed severely dilated left ventricle with severe systolic dysfunction (ejection fraction of 25%). The patient was assessed for heart transplantation and was put on heart transplantation waiting list. However, due to progressive clinical deterioration and hypo-perfusion related chronic renal failure, it was decided to bridge her to heart transplant with LVAD. Pre-operative echocardiography showed mild mitral stenosis (mean gradient of 4 mmHg and valve area of 2 cm^2^). This could have cause obstruction to flow across mitral valve and in turn inadequate flow through LVAD after implantation. Through median sternotomy, the patient was put on CPB. A circular piece of myocardium (diameter of 2 cm) at LV apex just lateral to left anterior descending artery was resected for insertion of HVAD (Heart Ware Inc, Framingham, MA) LVAD inflow cannula. Through this hole, mitral commissurotomy was performed by cutting open the previously repaired mitral valve, starting at centre and progressing to both commissures. In addition, primary chordae attached to anterior leaflet were also divided to offer complete opening of the valve. A HVAD was implanted through this hole in routine manner. Briefly, a metal ring was suture fixed around the hole and HVAD pump was implanted on LV through the ring. The outflow graft was anastomosed to ascending aorta using side biting clamp. The HVAD was started as CPB weaned off slowly to generate flows of 4 to 5 l/min. Postoperative echocardiography at discharge showed no gradient across mitral valve.

## Discussion

LVAD placement is now an accepted treatment for patients with end-stage heart failure [2,3]. Pre-existing native or prosthetic valve pathology does not increase the immediate perioperative risk of LVAD insertion; however, these patients continue to pose a challenge for postoperative management while awaiting transplantation [4]. Concomitant valve surgery during VAD implantation appears to be a reasonable option in end-stage heart failure patients with valvular heart disease [5]. Mitral stenosis causing obstruction to blood flow from left atrium to ventricle needs commissurotomy before LAVD implantation to ensure unobstructed flow to the LAVD. In the present case, due to previously repaired mitral valve potential flow obstruction was anticipated.

The conventional repair of mitral valve needs CPB and cardioplegic arrest of heart to open the left atrium. This imposes additional incision over left atrium, time consumption and potential source for bleeding especially with anticoagulation for LVAD. Additional CPB time, bleeding and transfusion may cause post LVAD right ventricular failure requiring temporary right ventricular assist. To avoid this, authors propose repair of mitral valve through the left ventricular apical hole which is created for LVAD inflow cannula.

Several approaches for mitral valve are mentioned in the literature, however, the commonly used are left atriotomy through the inter-atrial groove and trans-septal approach through the right atrium. Although, left atriotomy is more commonly used, trans-septal approach offers better mitral visualization in difficult cases especially small left atrium and re-do surgery [5]. The trans-septal approach is also useful in patients necessitating right atriotomy for concomitant procedures and it facilitates beating-heart mitral valve surgery, because retraction of the atrial wall during the trans-atrial approach often causes aortic insufficiency [6,7]. One of the scarcely used access for mitral valve is aortotomy. Through a conventional oblique aortotomy, the mitral valve can be repaired with techniques like neo-chorda implantation [8]. Trans-aortic edge-to-edge repair (Alfieri stitch) of the mitral valve along with minimally invasive aortic valve replacement can be performed to reduce the operative risk [9].

A trans-ventricular approach to mitral valve is ideally suited for patients having left ventriculotomy performed for left ventricular aneurysm [10]. The approach is useful for valve replacement or commissurotomy, although placement of an atrial mitral annuloplasty ring via this approach has been reported [11,12]. Trans-apical approach is further modification of trans-ventricular approach. Transcatheter mitral valve-in-ring implantation (TVIR) in high-risk patients after failure of surgical ring annuloplasty through LV apex has recently been reported [13]. The approach is also utilized to close mitral para valvular leakage using Amplatzer duct occlude [14]. Transapical beating heart chordae replacement for MV repair using the NeoChord device has also been introduced [15].

The apical orifice created to fit LVAD inflow is bigger than the one used for above mentioned catheter based procedures. Exposure through this apical orifice may not be sufficient exposure for mitral valve repair, but one can easily manage commissurotomy through it. This can be facilitated by bringing the orifice close to the mitral valve. In case of a narrowed prosthetic valve, the leaflets may be cut open through the apical orifice. In case of a small prosthesis or a doubtful commissurotomy, authors would prefer left atriotomy on beating heart (on-pump) and excision of prosthesis.

## Conclusions

Patients with end stage heart disease related to valvular disease should not be denied LVAD solely due to valvular disease. Mitral commissurotomy is feasible through an apical hole for LVAD inflow cannula.

## Consent

Written informed consent was obtained from the patients for publication of this Case report and accompanying images. A copy of the written consent is available for review by the Editor-in-Chief of this journal.

## Abbreviations

LVAD: Left ventricular assist device; CPB: Cardiopulmonary bypass.

## Competing interests

The authors declare that they have no competing interests.

## Authors’ contributions

PNM and NPP drafted the manuscript, BZ & SL analyzed and interpreted the patient data, AFP and MH conceptualize the manuscript, AS involved in the crucial revisions of manuscript, MA performed the surgery and participated in drafting the manuscript. All authors read and approved the final manuscript.
